# Tar in Skin Diseases

**Published:** 1875-07

**Authors:** 


Tar in Skin Diseases—It has [illegible]ong been known by old nurses throughout the country, and by thousands of mothers, that tar applied to the skin in cutaneous affections, proves decidedly curative in its influence. As the terebinthinate balsams are found to possess a specific influence over diseases of the mucous membrane so tar, which is similar in its medical properties to the fir and copaiba balsams, exerts a like power over diseases of the skin. Tar water, or water, slightly impregnated with tar by mixing and stirring has long been deemed valuable in weak stomachs and in throat affections. In fact, tar in various forms is believed to possess real healing power, as well as the power to eradicate many varieties of disease-germs. The trouble has been however that tar could not be applied to the skin without exciting emotions of disgust. In its natural state it stains the skin and renders it sticky and uncomfortable. The attempt has ofter been made to incorporate it with soap, but it has been discovered that very little pine tar can be so mingled with soap as to be retained. If a sufficient quantity of tar is added to produce its specific action upon a skin suffering from eruptions, it has been wont to exude and drain out of the soap. If only the small proportion was added which could readily be retained by the soap, it was so greatly diluted as to be practically inert. To devise a means by which tar could be saponified so as to become itself the actual and chief basis of the soap, was the subject of thousands of experiments, which were abandoned as futile.

At the movement cure of Dr. Geo. H. Taylor, on Madison Avenue and 58th street, New York, our attention was called to some cakes of "Packers Tar Soap," which Dr. Taylor spoke of in terms of real commendation. He seemed to think this article superior to any other of the kind, in use. We obtained a cake of it at a drug-store, and it speedily became a necessity in the family. Its cleansing qualities appear to be good; it forms an abundant lather; while the skin has a soft and pleasant feeling after its use, which other soaps do not occasion. Pimples and blotches and roughness disappear from the face under its influence, and all irritations are allayed and soothed.

In using this soap no indication is discovered of the existence of tar, except that of its pleasant aroma. You simply see a cake of fine transparent soap. But the fact is, this cake of soap is made chiefly of pure tar, completely saponified, by a process peculiar to Mr. Packer. With the finest pine tar and oil of cocoanut, acted upon by pure alkali, and steam pressure, he makes a pure, neutral soap, free from potash or animal fats, and possessing great healing qualities. As a toilet soap nothing can be nicer, while its reputation in eruptive diseases, whether in children or in adults appears to be firmly established.

